# Interpretable Framework for Sleep Monitoring: Applying Statistical Control Charts to Physiological Data Streams

**DOI:** 10.3390/s26123687

**Published:** 2026-06-09

**Authors:** Rupesh Agrawal, Dursun Delen, Bruce Benjamin

**Affiliations:** 1 Department of Health Informatics, College of Informatics, Northern Kentucky University, Highland Heights, KY 41099, USA; 2 Management Science and Information System, Spears School of Business, Oklahoma State University, Tulsa, OK 74106, USA; dursun.delen@okstate.edu; 3 Department of Management Information Systems, Faculty of Business Administration, Haliç University, Istanbul 34060, Türkiye; 4 Center for Health Sciences, Oklahoma State University, Tulsa, OK 74106, USA; bruce.benjamin2@gmail.com

**Keywords:** control charts, big data, streaming, analytics, anomaly detection, patient safety, decision support system

## Abstract

**Highlights:**

**What are the main findings?**

- Control charts reveal distinct and interpretable patterns of physiological variability across wake and non-REM sleep stages in polysomnography recordings.
- Cardio-respiratory signals exhibit greater variability during wakefulness and sleep stage transitions than during stable non-REM sleep.

**What are the implications of the main findings?**

- Control charts provide a transparent, rule-based method for exploring sleep-state dynamics in physiological time-series data.
- Control charts may serve as an interpretable baseline for sleep health monitoring and as a precursor or complementary method for future data-driven approaches.

**Abstract:**

Polysomnography monitors sleep health with non-linear physiological time-series data, consequently making interpretability a challenge. This study explores the feasibility of applying control charts, a statistical process control method, to cardio-respiratory signals derived from polysomnography studies to provide transparent and interpretable analysis of sleep-related physiological variability. Cardio-respiratory signals from a publicly available polysomnography dataset were preprocessed, transformed, and analyzed using univariate control charts. Sleep stage annotations were used as reference information to contextualize physiological variability across wake and non-REM sleep stages. Phase-level control chart rule violations were examined relative to annotated sleep-state transitions and summarized quantitatively. The results indicate that control chart rule violations occur more frequently during wakefulness and at wake–non-REM sleep transitions, while remaining relatively stable during sustained non-REM sleep. These findings indicate structural correspondence between SPC-based variability flags and annotated sleep stage dynamics. This exploratory, feasibility-focused study does not evaluate diagnostic performance or detection accuracy. Instead, it provides evidence that SPC control charts can serve as a transparent and interpretable analytical framework for exploring physiological variability in sleep data and for supporting future research on sleep-state analysis and explainable data-driven methods.

## 1. Introduction

With the rapid growth of patient health information (PHI) data, variability is inevitable [1]. Consequently, there is a need for interpretable methods for patient-centric diagnosis, treatment, and prognosis. Health Information Technology (HIT) supports the goals of the Agency of Healthcare Research and Quality (AHRQ) (https://www.ahrq.gov/cpi/index.html (accessed on 7 May 2026)) to improve the quality of diagnosis using patient information monitoring [2,3]. The human body is a complex adaptive system or “system of systems” [4,5]. Dynamic interacting systems, such as respiratory and circulatory systems, adapt to real-time stimulation such as weather, stress, and age to maintain homeostasis or a “normal” health state [6], such as normal temperature and blood pressure [7].

While control charts were introduced to solve manufacturing quality, they lend themselves precisely for monitoring and interpreting human health systems with rule-based, visually interpretable feedback [8]. Although the current study does not demonstrate automating, control charts in manufacturing are used for automating flagging anomalies without human intervention. Thus, control charts offer a rule-based, explicit, interpretable method in contrast with “black-box” methods such as Neural Network (NN) Machine Learning (ML) models. In the current work, control charts were evaluated to demonstrate an interpretable baseline to show visual monitoring of physiological signals. Future research may serve as a baseline reference and guide the extraction of structured features for desired explainable artificial intelligence (xAI) models and potential predictive performance.

Heart rate and airflow are preferred physiological signals in consumer wearable devices. To establish an absolute baseline signal for this study, it is derived from a “gold-standard” in-lab polysomnography (PSG) study to support future wearable settings. PSG represents a multimodal physiological process during sleep and characterizes the dynamics between discrete sleep stages. With the primary goal of clinical interpretability by annotating variability in non-stationary PSG signals, in this study, control charts are explored as a visual framework. The goal of the current study is to demonstrate the feasibility of interpretable SPC-based analytics as a foundation for future real-time and automated health anomaly monitoring for detection and prediction.

Accordingly, the contributions of the current feasibility study are threefold: (1) it demonstrates the steps for a phase-level framework for analyzing cardio-respiratory time-series aligned with clinically annotated sleep stages acquired from the PSG study, (2) it presents quantitative and visual evidence that SPC-based test violations preferentially occur during wakefulness and sleep stage transitions rather than stable non-REM sleep; and (3) it ascertains control charts as an interpretable method for exploring physiological variability, not as diagnostic method but as a precursor for a predictive model for sleep health anomalies.

### 1.1. Background

#### 1.1.1. History of Control Charts

The high cost of failure associated with underground telephone lines motivated the shift from quantity to quality manufacturing [9]. Dr. Walter Andrew Shewhart, who is called the father of SPC [10], introduced the concept between 1920 and 1924. SPC is a set of methods and tools developed to control (independent variable) and monitor (dependent variable) to evaluate the state of process control, where the process is a sequence of activities that transform inputs into outputs [11].

#### 1.1.2. Functions and Types of Shewhart Control Charts

Control charts are a core tool of SPC [12] and provide interpretable visual cues for routine (common-cause) and unusual (special-cause) variations around a centerline with boundary lines. For the current study, the control charts are used to show visual cardio-respiratory variability to identify common and special variations.

**Figure 1:**
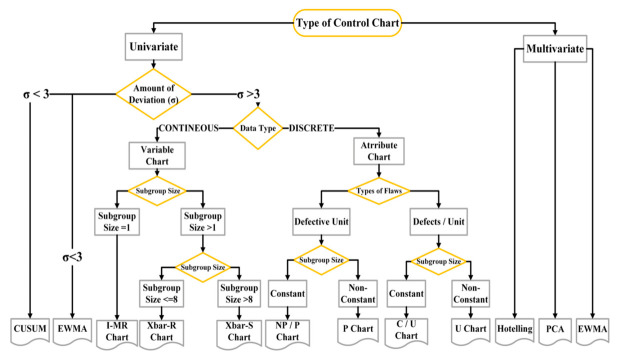
Classification of statistical process control chart types and their selection criteria, distinguishing univariate variable charts, univariate attribute charts, and multivariate charts.

There are three types of control charts: (a) variable charts, (b) attribute charts, and (c) multivariate charts. Variable and attribute charts are also called univariate charts. Please refer to Figure 1 for the types of control charts and the criteria for selection for each chart. Variable charts-mean (X^−^) and moving range (R) charts are the most-used control charts for moving data or time-series [13,14] to measure process states and variations in a system under observation.

**Figure 2:**
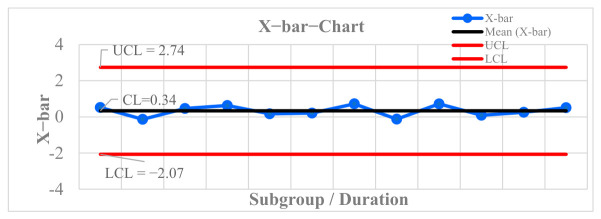

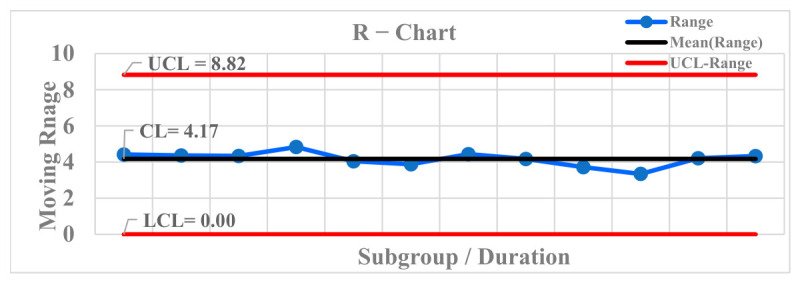
Example $\bar{X}$ and $\bar{X}\;-\;R$ control chart pair illustrating the concurrent visualization of subgroup means and within-subgroup variability.

The samples and subgroups for plotting the control charts are assumed to follow a normal distribution. Please refer to Figure 2, showing an example X-bar and X-bar-R chart with the CL, UCL, and LCL. Common control charts are always a set of two plots to show the mean (X-bar) and variability, such as the range (X-bar-R) or the standard deviation (X-bar-S).

#### 1.1.3. Application of Control Charts

The journey of SPC began as a framework for continuous quality improvement (CQI), enabling businesses to reduce manufacturing defects and costs and improve customer satisfaction [15,16,17,18,19]. Its cross-industry adoption demonstrated robustness towards total process control and improvement [20,21,22,23,24]. SPC’s interpretable, rule-based framework motivated its adoption in healthcare, transparent administration and evidence-based decision support without requiring complex modeling. In early stages, multidisciplinary clinics adopted SPC to improve delivery quality, reduce the length of stay, and lower the medication error rate [25,26,27,28,29,30]. Similarly, its (SPC) interpretability complemented novel adoption of pre-control chart checks to probe data bias using a time-series ARIMA model [31], and SPC-guided application led to a reduction in common infection [32].

#### 1.1.4. Real-Time Monitoring and Interpretable SPC-Based Analytics

Real-time health monitoring emerged in 1949 [33], and wider adoption began with the Holter monitor, which recorded the heart signal or electrocardiogram (ECG) over 24–48 h [34,35]. Advances in microelectronics and computing power have expanded wellness monitoring [36,37,38] using consumer wearables trackers and increased awareness of telemedicine [39]. However, remote diagnostic technologies embedded in consumer wearables (e.g., Fitbit) still require further development [36,40] for clinical acceptance. The ML model approach shows promise; however, the opaqueness of the decision pathway can limit clinical trust. In contrast, SPC offers rule-based transparency, which is a century-old approach with a proven track record. Accordingly, in this feasibility study, we propose using control charts as a visually interpretable baseline. The control charts provide visually readable flags to explain physiological variations (runs and trends). Control chart-derived flagged summaries can serve as clinical features for downstream ML to achieve the desired accuracy of lift.

### 1.2. Toward Interpretable and Automated Polysomnography Using Control Charts

#### 1.2.1. Overview and Motivation

The ability of control charts to identify variations or shifts in state (normal versus unhealthy) makes them a logical method for evaluating physiological signals that represent the human health process. Control charts spot or flag anomalies such as outliers and rare events via visual monitoring [8,41], and the ability to examine vital time-series signals with interpretable graphical visualization [42] offers real-time diagnostics with reduced error [43]. The current study explores control charts for establishing a framework that visualizes and flags anomalies in sleep stage transitions.

#### 1.2.2. Sleep Stages and Polysomnography

In an unhealthy adult, the sleep stage pattern and cycle deviate from those in a healthy state [44]. Understanding sleep stage patterns, especially how they vary across disease conditions, remains an active area of research, including questions of causality between sleep disruption and disease processes [44,45,46].

An electroencephalogram (EEG) is a collection of the brain’s electrical activity during sleep. Changes in EEG frequency characterize two types of sleep stages called rapid eye movement (REM) and non-REM. N1, N2, and N3 constitute non-REM sleep. Each sleep stage cycle lasts for approximately 90–120 min.

**Figure 3:**
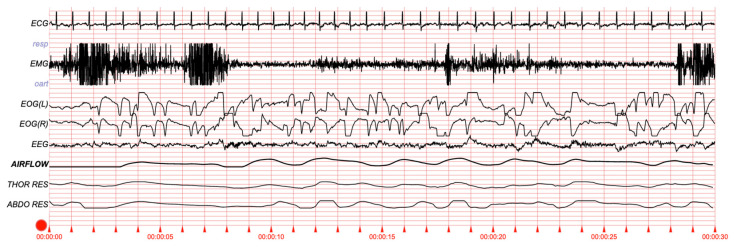
Example one-hour segment of polysomnography (PSG) data from the Sleep Heart Health Study (SHHS) adopted from archive.physionet.org (https://archive.physionet.org/lightwave/ (accessed on 7 May 2026)).

Overnight EEG, when collected with additional vital signals, such as electrocardiogram (ECG), electromyogram (EMG) (muscle movement), electrooculogram (EOG) (eye movements), blood oxygen levels (oxygen saturation), and nasal airflow (respiration), is called polysomnography (PSG). Polysomnography (PSG) is a non-emergency medical lab procedure for diagnosing a variety of sleep disorders, such as obstructive sleep apnea (OSA) and narcolepsy, which are diagnosed via PSG evaluation [47]. Figure 3 illustrates a one-hour segment of PSG acquired from the Sleep Heart Health Study (SHHS) [48].

#### 1.2.3. Interpretable Automation in Polysomnography

The current PSG system performs initial automated sleep staging and scoring. However, automated evaluations are manually reviewed by a registered polysomnography technician (RPSGT) to diagnose sleep disorders or a patient’s health [47]. In the current study, PSG serves as a reference for contextualizing physiological variability, particularly cardio-respiratory patterns.

The emergence of remote health monitoring via ubiquitous wearable devices and cumbersome EEG sensors placement has motivated research to explore the use of other physiological signals, such as ECG and respiration, for sleep health evaluation [49,50]. The current adoption and availability of real-time data using wearable devices is motivating automating the diagnostic process for clinical decision support [51,52]. Contemporary research and commercial efforts have demonstrated the ability to predict health conditions such as atrial fibrillation (AF) and OSA using wearable devices [53,54,55,56]. The primary approaches used in many of these solutions rely on ML models, with an emphasis on predictive accuracy, whose internal decision pathways may be difficult for clinical interpretations.

The primary goal of this study is to demonstrate the use of control charts as an interpretable monitoring framework for PSG-derived physiological signals. The goal of this research work is to show rule-based criteria for explicit automated health anomaly flagging, similar to that achieved by control charts in other domains, such as manufacturing.

Extant research studies have pointed toward the use of control charts in health and healthcare studies. However, to the best of our knowledge, the literature review did not identify work with a ground-up approach for the use of control charts in identifying and evaluating sleep/stages and abnormalities/diseases using PSG signals such as heart rate and respiration signal data. Thus, the current study presents an evaluation of control charts as a transparent baseline to visualize cardio-respiratory variability for annotating sleep stage transitions. In future research, the foundational framework may serve as structured features for machine-learning models, for explainable AI (xAI) and for potential improvements in predictive performance.

## 2. Materials and Methods

### 2.1. The Nature of Sleep Data

The current study utilized a PSG dataset acquired from SHHS to determine the cardiovascular consequences of sleep-disordered breathing [48] and was analyzed using Tableau (version 2026.1.1, Seattle, WA 98103, USA). PSG data from a single subject, ID #0000, a 47-year-old individual with a BMI of 29.73, was used for the analyses. Table 1 details the name of the signal, notation, data collected per second for each signal, and frequency of each signal.

### 2.2. Signal Selection and Justification

The final selection of heart rate (HR) and nasal airflow signal was motivated by their relevance in clinical evaluation, adoption, accessibility, and explainability. Although EEG remains the gold standard for sleep staging, signal selection was deliberately constrained to well-established cardio-respiratory physiological measures to focus on validating the feasibility of control chart application to PSG data under known sleep stage annotations. The American Thoracic Society recommends cardio-respiratory signals for evaluating sleep studies [57,58] and for diagnosis of low-risk and high-risk conditions, such as stress [59] and congestive heart failure [60]. Airflow measurements are indicative of OSA diagnosis [47] and are the primary signal recommended for use in HST by the American Academy of Sleep Medicine (AASM) [61].

Cardio-respiratory changes occur within and during each sleep stage transition. HR typically decreases across successive sleep stages, reaching the lowest value in the non-REM (N3) stage and its highest value in REM sleep. Similarly, respiration is shallower across successive sleep stages, with the lowest measurement in the REM stage [62]. Additionally, disease conditions accentuate cardio-respiratory variability [57,62]. Finally, both HR and respiration sensors are widely available, affordable, and clinically validated for remote monitoring [63,64].

### 2.3. Method

#### 2.3.1. SPC Terminology and Control Chart Interpretation Tests

This section explains the preprocessing steps and subgrouping strategy, with a focus on the reproducibility and interpretability of control charts to demonstrate the feasibility of their study. The following notations and terminology are provided to demonstrate the application of control charts [65]. In this study, the variables analyzed using control charts were HR and airflow, each treated independently as a univariate physiological signal.

The Western Electric tests are standard control chart guidelines, applied in this study to support rule-based and interpretable visual monitoring [66]. These tests, which were simple and guided the analysis of the results reported in Section 3, were as follows: Test 1: One or more points falling outside the ±3σ control limits (UCL or LCL) indicate a large shift in the process mean. Test 2: Seven (7) or more consecutive points occurring on the same side of the centerline (CL) indicate a sustained shift in the process mean. Test 3: Six (6) or more consecutive points exhibiting a monotonic increase or decrease indicate a trend in the process.

Within each subgroup (N), the sample mean ($\bar{X})$ (Equation (1)) is computed as the average of the observations, and the range (R) (Equation (2)) is calculated as the difference between the maximum and minimum values, capturing the short-term dispersion of the physiological signal. The process means ($\overset{̿}{X})$ (Equation (3)) is defined as the average of the subgroup means. In addition, the moving range (MR) (Equation (4)) is calculated as the absolute difference between two consecutive observations in the time-series and is used to quantify point-to-point variability in HR and airflow signals. The mean moving range ($\bar{MR}$) (Equation (5)) represents the average of individual moving ranges, whereas the mean range ($\bar{R}$) (Equation (6)) summarizes within-subgroup variability and is used for estimating control limits in ($\bar{X}-R)$ charts.

(1) $$\bar{X}=\sum \frac{\left(x_{i}\ldots x_{n}\right)}{n}$$

(2) $$R=x_{max}-x_{min}$$

(3) $$\overset{̿}{X}=\sum \frac{{\bar{X}}_{i}\ldots {\bar{X}}_{N}}{N}$$

(4) $$R=\left|x_{i}-x_{i-1}\right|$$

(5) $$\bar{MR}=\frac{\sum \left({MR}_{i}\ldots {MR}_{n}\right)}{\left(n-1\right)}$$

(6) $$\bar{R}=\sum \frac{\left|{\bar{R}}_{i}\ldots {\bar{R}}_{N}\right|}{N}$$

The within-subgroup standard deviation ($s_{i}$) (Equation (7)) quantifies dispersion of observations around the subgroup mean, and the average subgroup standard deviation ($\bar{s}$) (Equation (8)) summarizes this variability across subgroups. The process standard deviation (σ) (Equation (9)) is estimated from $\bar{s}$ using the SPC constant c_4_ (refer to Table 2 for the value of the constant) and is used in computing control limits for $\left(\bar{X}-S\right)$ charts, where subgroup variability is characterized by the standard deviation rather than the range.

(7) $$s_{i}=\sqrt{\frac{{\left({\bar{x}}_{i}-\bar{X}\right)}^{2}}{n}}$$

(8) $$\bar{s}=\frac{\sum \left(s_{i}\ldots s_{n}\right)}{N}$$

(9) $$\sigma =\frac{\bar{s}}{c_{4}}$$

The above summary statistics were used to estimate the control limits for the control charts for subsequent use in this study. For the individuals–moving range (I−MR) chart, the individual-chart centerline (${CL}_{I}$) corresponds to the process mean $\left(\bar{X}\right),\;$where the control limits (${{LCL}_{I},\;UCL}_{I})$ are estimated from the ($\bar{MR}$) and SPC constants (Equations (10) and (11)). Similarly, the moving-range chart limits are derived from using Equations (12) and (13). The centerline (CL) and upper and lower control limits $\left(LCL,UCL\right)\;$are shown in Figure 2, where the SPC constants are adopted from the Institute of Quality and Reliability and are tabulated in Table 2. The constants for control charts are as follows.

I-moving range (MR):

(10) $${\frac{UCL}{LCL}}_{I}=\bar{X}+/-3\frac{\bar{MR}}{D_{2}}$$

(11) $${CL}_{I}=\bar{X}$$

(12) $${\frac{UCL}{LCL}}_{MR}=\bar{MR}+-3D_{3}\frac{\bar{MR}}{D_{2}}$$

(13) $${CL}_{MR}=\bar{MR}$$

For $\left(\bar{X}-R\right)$ charts, the control limits for subgroup means are calculated using the process mean $\left(\overset{̿}{X}\right)\;$and the average subgroup range ($\bar{R}$) (Equation (14)), and the range chart limits are calculated using Equations (15)–(17).

($\bar{X}$)–range (*R*):

(14) $${\frac{UCL}{LCL}}_{xR}=\overset{̿}{X}+/-A_{2}\bar{R}$$

(15) $${CL}_{xR}=\overset{̿}{X}$$

(16) $${UCL}_{R}=D_{4}\bar{R}$$

(17) $${LCL}_{R}=D_{3}\bar{R}$$

(18) $${CL}_{R}=\bar{R}$$

For $\left(\bar{X}-S\right)$ charts, the mean chart limits are computed using the process mean $\bar{X}$ and estimated process standard deviation (σ) (Equations (19) and (20)), and the standard deviation chart limits are calculated from the average subgroup standard deviation (Equations (21) and (22)). These limits are data-derived control limits to assess departures from statistical control and to generate qualitative variability flags, rather than clinical or specification thresholds.

($\bar{X}$)–std. deviation (σ):

(19) $${\frac{UCL}{LCL}}_{xS}=\overset{̿}{X}+/-3\sigma$$

(20) $${CL}_{xS}=\overset{̿}{X}$$

(21) $${\frac{UCL}{LCL}}_{xS}=\bar{s}+/-3\sigma$$

(22) $${CL}_{xS}=\bar{s}$$

#### 2.3.2. Sample, Subgroup, and Phase Definition

The SPC guidelines introduced in Section 2.3.1 guided the current section for the selection of observations (samples) for constructing rational subgroups that preserved within-subgroup homogeneity and between-subgroup variability to reflect process shifts [65,66,67,68]. In this study, each sample corresponds to a single time-ordered physiological measurement, such as one second of HR or airflow, whereas the sample size (n) denotes the number of observations within each subgroup, while the subgroup size (N) refers to the total number of subgroups evaluated [65,66].

The PSG data were clinically scored using 30 s epochs, with each epoch assigned a discrete sleep stage [47]; thus, each epoch corresponded to sleep stage transitions and preserved visual alignment with annotated stage boundaries. A heuristic subgroup size of *n* = 10 observations (10 s) was selected for optimal temporal resolution and to align with 30 s epochs. This subgroup definition resulted in *n* = 6 subgroups per minute-phase for the assessment of variability within a sleep stage and across sleep stage transitions. The subgroup was designed for temporal interpretability and alignment with clinical sleep scoring only, and not for optimization of statistical power.

Control chart center lines and limits were computed using within-subject, phase-level subgroup statistics, such that the averages, ranges, and standard deviations reflected each subject’s own physiological variability rather than pooled cohort-level reference distributions. Consequently, the control chart signals should be interpreted as relative indicators of variability for the analyzed subject or patients’ recordings, rather than a healthy patient population baseline.

Wake segments not adjacent to sleep stage transitions were excluded to focus on within-subject physiological variability associated with dynamic sleep state changes only. Prior to control chart construction, the normality assumption of the data was tested to comply with the application of univariate variable control charts, and this is elaborated on in Section 2.3.3.

#### 2.3.3. Control Charts’ Selection and Rationale

SPC provides two types of families of control charts, as shown in Figure 1, designed to monitor process behavior and detect departures from statistical control based on the nature of the data and the monitoring objective. Chart types vary in their sensitivity to large shifts, sustained mean shifts, and short-term variability, making chart selection a critical methodological decision [13,14].

In the current study, three commonly used univariate control charts were evaluated: Individuals–Moving Range (*I–MR*), Mean ($\bar{X}$) and Range Chart ($\bar{X}$ − *R*), and Mean ($\bar{X}$) and Standard Deviation Chart ($\bar{X}-S$). I-MR charts are useful when subgroup sizes are small and evaluate individual observations around the process mean and quantify point-to-point variability using the MR. $\bar{X}$ and $\bar{X}$ − *R* charts are recommended for subgroup sizes between two and ten to monitor subgroup means around the overall process mean while using the range (R) to characterize within-subgroup dispersion. $\bar{X}$ and $\bar{X}-S$ charts are similar to $\bar{X}$ and $\bar{X}$ − R charts, but estimate dispersion using the subgroup standard deviation (S) rather than the range (R). An $\bar{X}-S$ chart is used for a large sample size and subgroup size of ten or higher.

Univariate charts, as the name suggests, use one variable to characterize process stability, variability, and shifts in the continuous time-series physiological signals [13,14,65]. Although all variable charts use continuous data, each control chart uniquely reviews and explains the process under examination. Control charts are usually applied in pairs to monitor a given process, where the first chart monitors the central tendency, and the companion chart monitors dispersion.

#### 2.3.4. Data Selection, Cleaning, and Transformation

**Figure 4:**
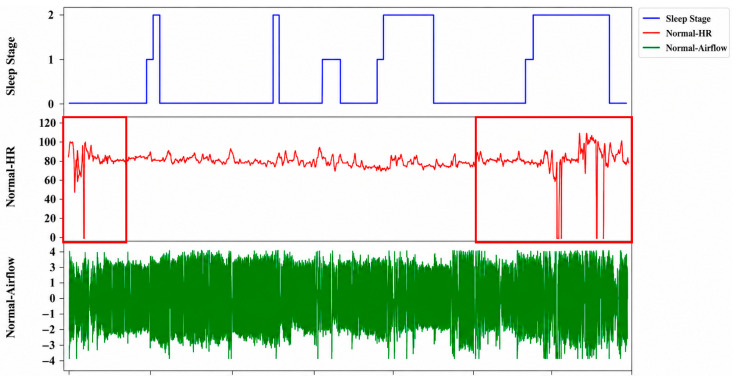
Sixty–minute segment of PSG–derived time-series data of sleep stage, heart rate, and nasal airflow extracted from an overnight recording showing variability across sleep states.

Visual analysis of approximately six hours of PSG data was conducted for data preprocessing and selection. For methodological transparency in handling, manipulation, and transformation, 60 min of the physiological time-series shown in Figure 4 were extracted for analysis. Signal artifacts or noise were identified and removed through visual inspection prior to analysis. Extended wake periods not relevant to sleep stage transition analysis were excluded to focus the evaluation on physiologically meaningful variability across sleep states. To ensure temporal consistency across physiological signals recorded at different sampling frequencies, as shown in Table 1, HR (125 Hz) and respiratory signals (10 Hz) were resampled and averaged to a common 1 Hz time scale.

**Figure 5:**
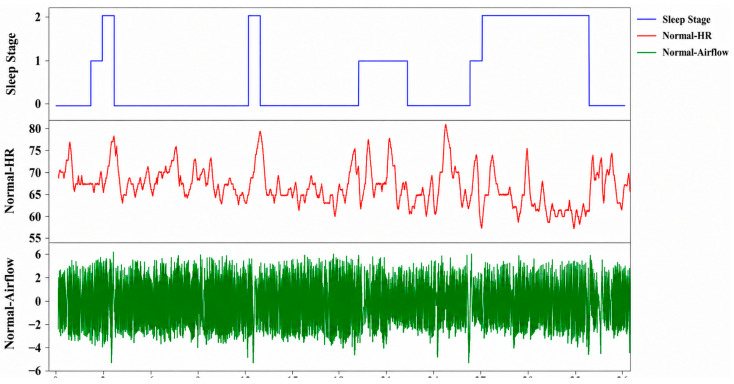
Physiological time–series signal recording for the twenty–seven–minute interval. This segment was extracted following noise removal.

**Figure 6:**
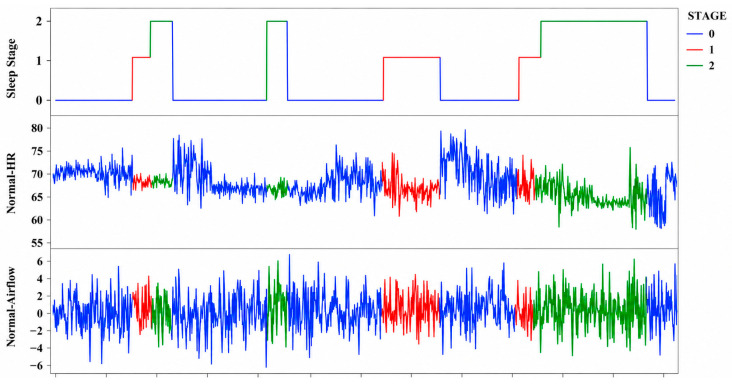
Corresponding sleep stage annotations for the seventeen-minute analysis interval after removing long-wake-phase data.

Following the noise removal, data between 8 and 34 min (twenty-seven-minute interval) were extracted, containing both wakefulness and non-REM sleep stages. Figure 5 illustrates the extracted physiological signals. The data were further refined, and long-wake-phase data were removed, such as data at 12–18, 22, and 27 min, to maximize variability in the final seventeen-minute time-series window. Figure 6 presents the corresponding sleep stage annotations. The associated minute-level phase-level data details are summarized in Table 3 and were used in the final analysis, resulting in 1020 data points per signal.

**Figure 7:**
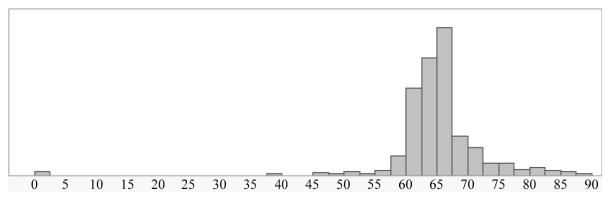
Distribution histograms of raw (0–60 min) HR prior to artifact removal, illustrating deviations from normality assessed using visual inspection and the Shapiro–Wilk test.

**Figure 8:**
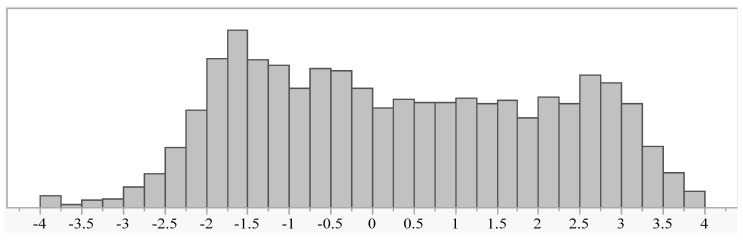
Distribution histograms of raw (0–60 min) airflow signal prior to artifact removal, illustrating deviations from normality assessed using visual inspection and the Shapiro–Wilk test.

The univariate control charts assumed approximate normality within subgroups; thus, the HR and airflow signals were evaluated for normality using visual inspection and the Shapiro–Wilk normality test [69]. Distribution histograms and goodness-of-fit results for the raw signals are shown in Figure 7 and Figure 8.

**Figure 9:**
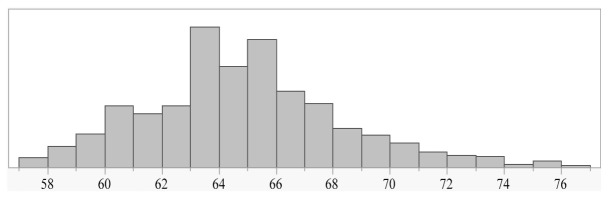
Distribution histograms of HR signals (twenty-seven minutes) after artifact removal and temporal alignment, and distributional characteristics following preprocessing.

**Figure 10:**
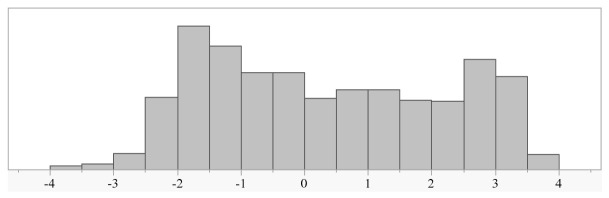
Distribution histograms of nasal airflow signals (twenty-seven minutes) after artifact removal and temporal alignment, and distributional characteristics following preprocessing.

Following noise removal, the distributional characteristics of the cleaned signals (twenty-seven minutes) were reassessed (Figure 9 and Figure 10).

Table 4 shows a summary of the relevant statistics of the sample distribution. The goodness-of-fit test resulted in *p* < 0.05 for HR and *p* > 0.05 for airflow data, which led to the conclusion that the dataset for HR did not come from a normal population; however, airflow data was normal after noise removal.

**Figure 11:**
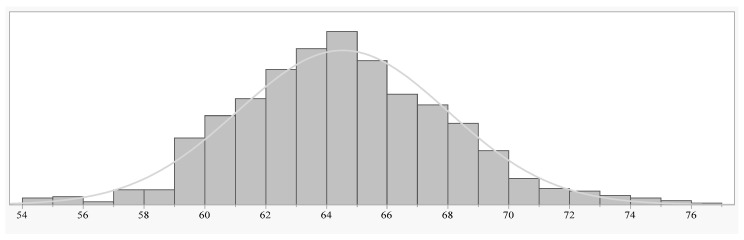
Log-transformed distribution of HR signals after preprocessing to illustrate changes in distributional shape.

**Figure 12:**
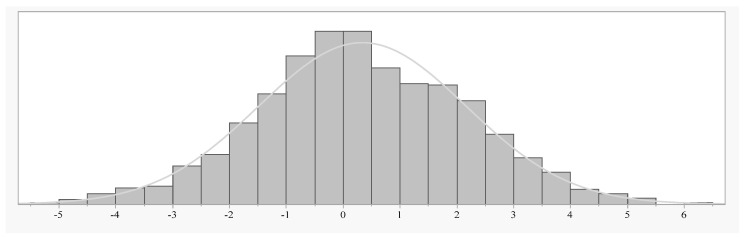
Log-transformed distribution of nasal airflow signals after preprocessing to illustrate changes in distributional shape.

Consequently, additional transformation using log-normal transformation, consistent with SPC practice for non-normal data, was conducted to ensure that the final data for the harmonized subgroups followed a normal distribution and *p*-value > 0.05 (refer to Figure 11 and Figure 12). A log-normal transformation was applied to both HR and nasal airflow signals to maintain methodological consistency across analyses. To assess distributional conformity at the phase level, Shapiro–Wilk goodness-of-fit tests were computed independently for each phase. The phase-level test results for the transformed signals are summarized in Table 5, indicating no statistically significant departures from normality at the chosen significance level.

To assess distributional conformity, phase-level Shapiro–Wilk goodness-of-fit *p*-values were combined using Fisher’s method [70], which aggregates independent tests into a chi-square-distributed statistic. The combined results for the log-transformed HR and nasal airflow signals are summarized in Table 6. The aggregated tests did not indicate statistically significant departures from normality across the final analysis window. This assessment supports the use of control charts for exploratory analysis of physiological signals.

## 3. Results

### 3.1. Objective and Summary of Rule Test

The objective of the Section 3 is to test and establish the feasibility of control chart rule violations in cardio-respiratory signals to align with physiologically meaningful changes in sleep state, and not to establish the diagnostic accuracy of health anomalies. In the current study, sleep stage annotations from polysomnography were used to contextualize variability patterns across wake and non-REM sleep stages.

The application of control charts to physiological time-series data was conducted in four steps: (a) visual inspection of raw data (0–60 min); (b) artifact removal and temporal smoothing (8–34 min); (c) a test for normality and subsequent transformation of signal data to satisfy normality conditions, and finalizing the datasets, which represented physiologically meaningful and statistically valid inputs for control chart interpretation; and (d) application of Western Electric tests at the phase (one-minute) level and interpreted relative to annotated sleep stages and stage transitions.

The four steps enabled rule-based structural assessment to preferentially flag variability within and across sleep stage transitions, thereby supporting the potential utility of control charts for interpretable sleep state and anomaly monitoring.

Table 7 summarizes the SPC rule violations across wake and non-REM sleep phases for HR and airflow signals. For both physiological signals, rule violations occurred more frequently during wake phases than during stable non-REM sleep. HR signals exhibited the highest percentage (75%) of flagged phases in wake-state SPC rule violation, compared with 11.1% during non-REM sleep. Airflow signals demonstrated a similar but less pronounced pattern, with 50% of wake phases flagged versus 11.1% during non-REM sleep.

### 3.2. Sleep Stage Variability and Rule Test Violations

The above results indicated that physiological variability is present in dynamic sleep states and transitions. Across both physiological signals, SPC rule violations were observed more frequently during the wake state and at wake–non-REM transitions than during relatively stable non-REM sleep. Although this pattern was less pronounced for airflow signals, for both signals, the majority of flagged phases occurred during wakefulness or immediately adjacent to sleep stage transitions, whereas only a small proportion of non-REM phases exhibited rule violations. Collectively, these findings support the potential utility of SPC-based control chart rules for interpretable monitoring of sleep state changes by preferentially flagging physiological variability during dynamic sleep states and transitions.

### 3.3. Representative Control Chart Examples

The current section presents representative control chart examples to illustrate how SPC-based variability flags manifest in physiological time-series data. The figures provide evidence to support the interpretability and visual demonstration of typical patterns of variability observed during wakefulness, non-REM sleep, and sleep stage transitions.

**Figure 13:**
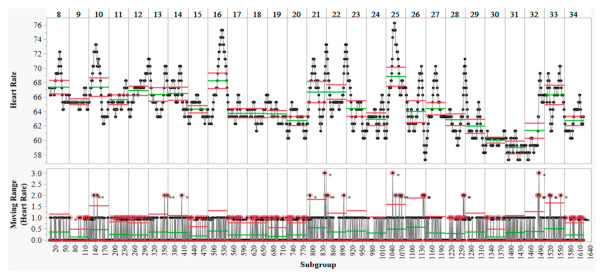
Individual–Moving Range (I–MR) control chart for raw heart rate signals (8–34 min interval), showing unstable variability before transformation.

**Figure 14:**
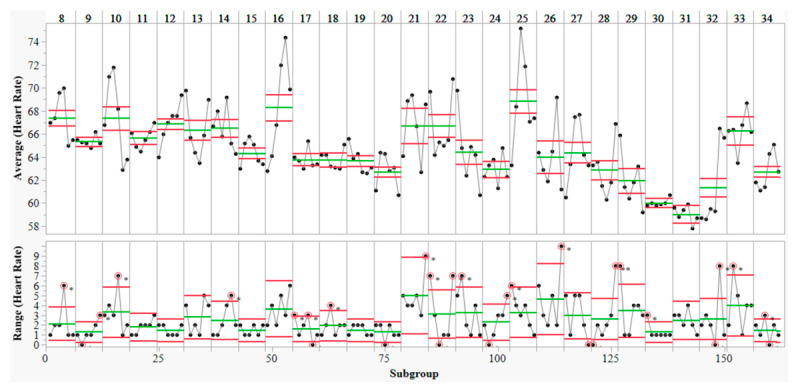
$\bar{X}$-R control chart for raw heart rate signals (8–34 min interval), showing mean values beyond control limits and unstable subgroup ranges, which indicate within-group variability.

**Figure 15:**
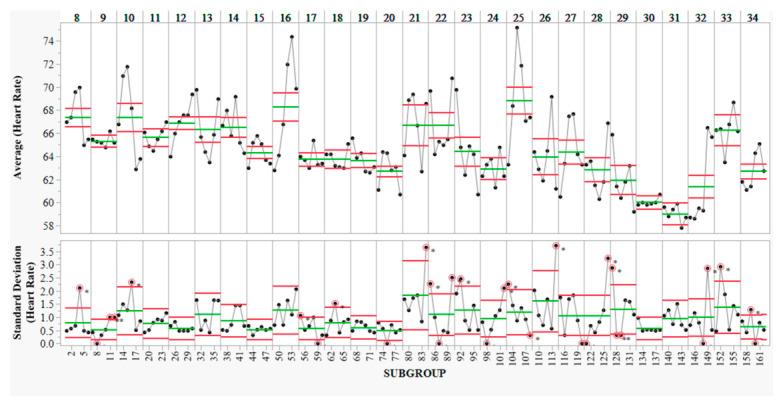
$\bar{X}$-S control chart for raw heart rate signals (8–34 min interval), showing elevated and unstable subgroup standard deviations that highlight the limitations of directly applying SPC methods to untransformed physiological signals.

Figure 13, Figure 14 and Figure 15 present representative I–MR, X^−^–R, and X^−^–S control chart examples for HR signals prior to transformation. Red circled points indicate out-of-control points that violate one or more control-chart tests. Stars (*) indicate the specific observation where the alarm was detected. Thus these charts exhibit frequent rule violations and unstable dispersion, consistent with the non-normal distribution of the raw HR data, illustrating the limitations of directly applying SPC methods to untransformed physiological signals.

**Figure 16:**
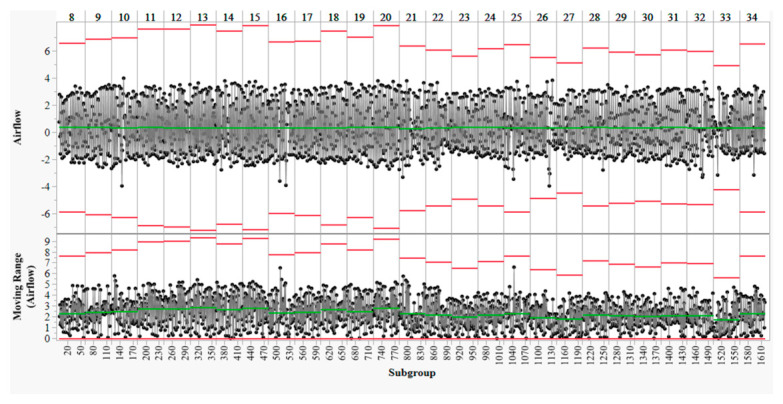
Individual–Moving Range (I–MR) control chart for nasal airflow signals (8–34 min interval), showing intermittent unstable variability before transformation.

**Figure 17:**
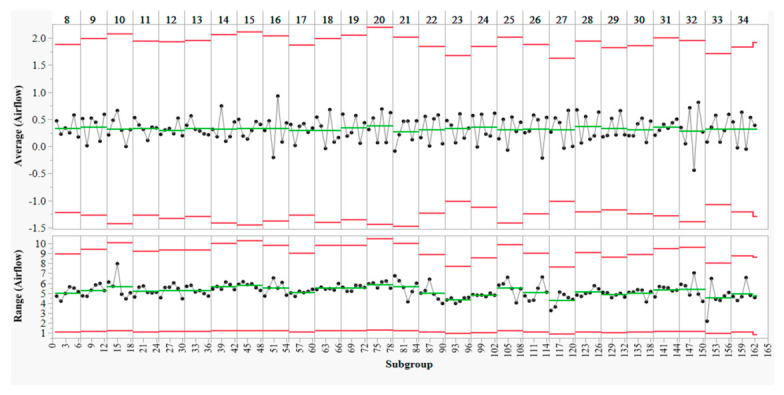
$\bar{X}$-R control chart for nasal airflow signals (8–34 min interval), showing mean values occasionally beyond control limits and intermittent elevated subgroup ranges.

**Figure 18:**
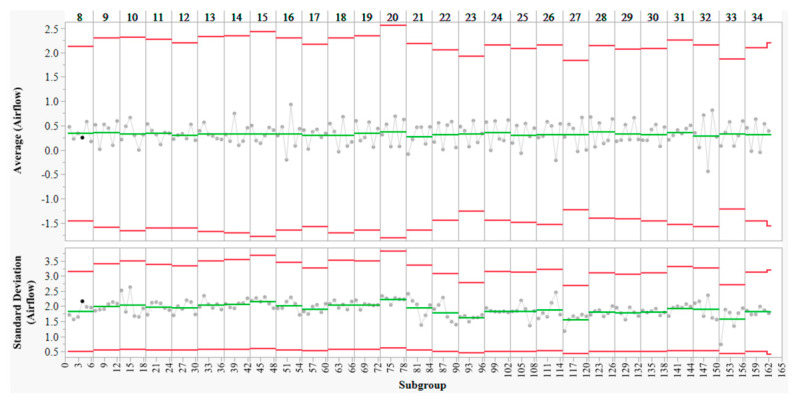
$\bar{X}$-S control chart for nasal airflow signals (8–34 min interval), showing occasional unstable subgroup standard deviations.

Figure 16, Figure 17 and Figure 18 show representative control chart examples for airflow signals prior to transformation. Similar to HR signals, airflow signals exhibit non-normal variability and intermittent rule violations, confirming the limitations of directly applying SPC methods to untransformed physiological signals; however, airflow exhibits relatively greater stability compared with HR signals.

**Figure 19:**
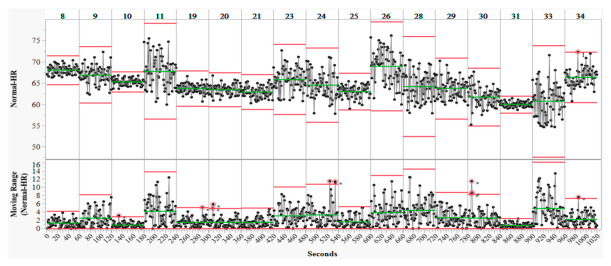
Individual–Moving Range (I–MR) control chart for log-transformed, approximately normal heart rate signals over the final analysis window.

**Figure 20:**
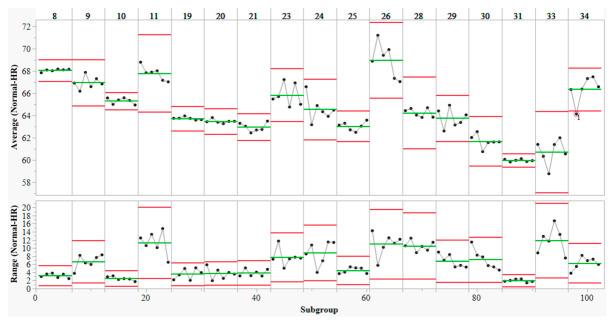
$\bar{X}$-R control chart for log-transformed, approximately normal heart rate signals across subgroups within the final analysis window, showing stability of mean values and ranges during non-REM sleep.

**Figure 21:**
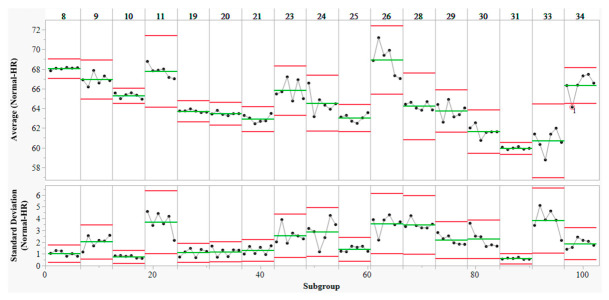
$\bar{X}$-S control chart for log-transformed, approximately normal heart rate signals across subgroups within the final analysis window, showing stability of subgroup standard deviations during non-REM sleep.

Figure 19, Figure 20 and Figure 21 present representative control chart examples for transformed-normal HR data. In alignment with the quantitative results reported earlier, rule violations in these charts are concentrated during wake phases and near wake–non-REM transitions, with reduced variability observed during stable non-REM sleep.

**Figure 22:**
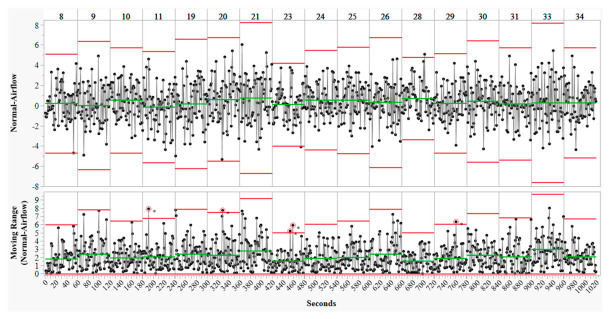
Individual–Moving Range (I–MR) control chart for log-transformed, approximately normal nasal airflow signals over the final analysis window.

**Figure 23:**
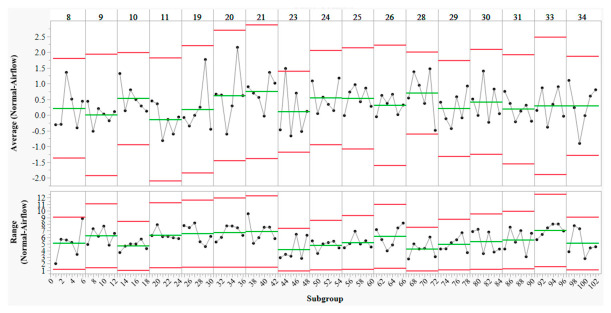
$\bar{X}$-R control chart for log-transformed, approximately normal nasal airflow signals across subgroups within the final analysis window, showing stability of mean values and ranges during non-REM sleep.

**Figure 24:**
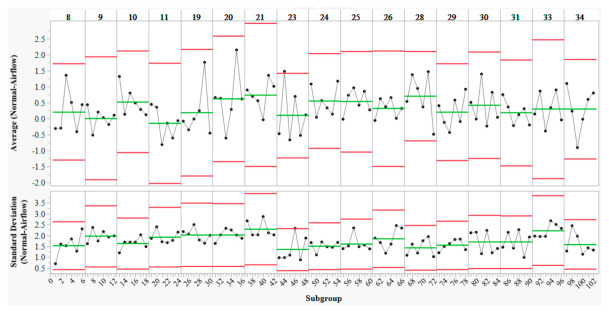
$\bar{X}$-S control chart for log-transformed, approximately normal nasal airflow signals across subgroups within the final analysis window, showing stability of subgroup standard deviations during non-REM sleep.

Figure 22, Figure 23 and Figure 24 illustrate representative control chart behavior for transformed-normal airflow signals. These charts demonstrate reduced dispersion and fewer rule violations during non-REM sleep phases, further supporting the interpretability of SPC-based monitoring after appropriate preprocessing.

Collectively, these representative examples visually reinforce the quantitative findings that SPC-based control chart rules flag physiological variability during dynamic sleep states and transitions, while remaining relatively stable during sustained non-REM sleep.

## 4. Discussion

### 4.1. Findings’ Interpretation and Inference

This study was designed to demonstrate the feasibility of visual evaluation to test SPC-based control chart rules for cardio-respiratory time-series data aligned with dynamic physiological sleep states. The results demonstrate the applicability of SPC, summarizing that violations preferentially occur during wakefulness and at wake–non-REM transitions, while remaining relatively stable during sustained non-REM sleep. These findings indicate structural alignment between SPC-based variability flags and annotated sleep stage dynamics. As per the study objective, the current results do not establish detection of health anomalies or predictive value for detecting obstructive sleep apnea or other sleep disorders. Accordingly, the findings are evidence of methodological feasibility and interpretability, rather than a clinical detection system.

### 4.2. Interpretability and Explainable AI

A key contribution of this work lies in demonstrating the potential role of control charts as a transparent and interpretable method for analyzing non-linear physiological time-series data in sleep research. Compared with “black-box” AI-ML models, SPC methods provide explicit, rule-based guidelines for readily inspecting dynamic variability for clinical comprehension. The results suggest that SPC may serve as a complementary framework for characterizing physiological variability, guiding feature extraction, or supporting explainable model development for data-driven predictive models.

### 4.3. Methodological Implications and Limitations

This study emphasizes several methodological considerations for control charts’ application to physiological signals. The methodological considerations emphasize the importance of data preprocessing, defining subgroups, and contextual interpretation when adapting SPC methods from industrial settings to biomedical time-series analysis.

First, identifying and removing artifacts requires careful preprocessing, as direct application to raw, non-normal physiological data can yield unstable or misleading signals and affect subgroup-level normality assumptions. Second, phase-level aggregation helps with clinically meaningful sleep annotations and provides a practical compromise between temporal resolution and interpretability.

Although the current study presents a transparent and interpretable solution for sleep research, this study has several limitations. The analysis was conducted in a limited time window in a single subject, and to generalize the results to full-night recordings or broader populations, further research is mandatory. SPC rule violations were evaluated only for sleep stage annotations and not against cardio-respiratory events such as apnea or hypopnea. Additionally, this study requires comparing SPC-based annotation with alternative detection performance metrics.

### 4.4. Future Research

Future work should extend this feasibility analysis by evaluating larger cohorts, multivariate control charts, and full-night or extended PSG studies. A broader scope is needed for quantifying detection performance against annotated cardio-respiratory events and comparing SPC outputs with other methods, such as machine learning approaches. Future research shall strengthen the robustness, clinical relevance, and potential clinical decision support.

## 5. Conclusions

The current research work evaluated the feasibility of statistical process control (SPC) by demonstrating the application of control charts to physiological time-series data for interpretable monitoring of sleep state dynamics. Using HR and airflow signals from a publicly available PSG dataset, the analysis demonstrated that SPC-based control chart rule violations preferentially occur during wakefulness and at wake–non-REM sleep transitions, while remaining relatively stable during sustained non-REM sleep. These findings indicate structural alignment between SPC-based variability flags and annotated sleep stage dynamics.

The importance of the current work is methodological scope rather than diagnostic prediction. This study provides evidence that SPC-based control charts can serve as a transparent and interpretable analytical framework for exploring physiological variability in sleep data and for contextualizing dynamic changes across sleep states, and may lend itself as a precursor or complementary method for improving accuracy and explainability for AI models.

Overall, this feasibility study for demonstration and evaluation highlights the potential role of SPC as an interpretable method for sleep state monitoring and as a complementary tool for future data-driven approaches. Further research involving larger cohorts, full-night recordings, and quantitative validation against annotated respiratory events is required to assess robustness, generalizability, and clinical relevance.

## Figures and Tables

**Table 1:** Polysomnography signal data summary.

Name	Notation	Frequency
Oxygen saturation	SaO2	1 Hz
Heart rate	HR	1 Hz
Electrocardiogram	ECG	125 Hz
Electromyogram	EMG	125 Hz
Electrooculogram	EOG(L)	50 Hz
Electrooculogram	EOG(R)	50 Hz
Electroencephalogram	EEG	125 Hz
Respiration (nasal)	AIRFLOW	10 Hz
Respiration (thoracic)	THOR RES	10 Hz
Respiration (abdominal)	ABDO RES	10 Hz

**Table 2:** Standard statistical process control (SPC) constants A2, D2, D3, D4, and C4 as a function of subgroup size (N), used for construction of control charts.

Subgroup Size (N)	A2	D2	D3	D4	C4
2	1.880	1.128	-	3.267	0.798
3	1.023	1.693	-	2.574	0.886
4	0.729	2.059	-	2.282	0.921
5	0.577	2.326	-	2.114	0.940
6	0.483	2.534	-	2.004	0.952
7	0.419	2.704	0.076	1.924	0.973
8	0.373	2.847	0.136	1.864	0.982
9	0.337	2.970	0.184	1.816	0.990
10	0.308	3.078	0.223	1.777	0.798
15	0.223	3.472	0.347	1.653	0.886
25	0.153	3.931	0.459	1.541	0.921

Adopted from Table of Constants for Control Charts–Institute of Quality and Reliability.

**Table 3:** Minute-level phase-level summary of physiological signals and associated sleep stage labels for the selected seventeen-minute analysis interval used in the final evaluation.

Phase	Sleep Stage
8 min	Wake (0)
9 min	Wake (0)
10 min	Non-REM (1)
10.5 min	Non-REM (2)
11 min	Wake (0)
19 min	Wake (0)
20 min	Wake (0)
20.5 min	Non-REM (2)
21 min	Wake (0)
23 min	Wake (0)
24 min	Wake (0)
24.5 min	Non-REM (1)
25 min	Non-REM (1)
26 min	Wake (0)
28 min	Wake (0)
29 min	Non-REM (1)
29.5 min	Non-REM (2)
30 min	Non-REM (2)
31 min	Non-REM (2)
33 min	Non-REM (2)
33.5 min	Wake (0)
34 min	Wake (0)

**Table 4:** Summary of Shapiro–Wilk normality test results for raw HR and airflow signals, indicating statistically significant deviations from normality (*p* < 0.05).

Summary Statistics	Heart Rate (Raw)	Heart Rate (w/o Noise, Long Wake)	Airflow (Raw)	Airflow (w/o Noise, Long Wake)
Mean	65.15	64.55	0.32	0.34
Std. dev.	7.77	3.45	1.80	1.83
Upper 95% mean	65.41	64.76	0.38	0.46
Lower 95% mean	64.90	64.33	0.26	0.23
N	3600	1020	3600	1020
Goodness of fit (*p*-value)	0.0001	0.0005	0.0001	0.6807

**Table 5:** Phase-level Shapiro–Wilk goodness-of-fit results for log-transformed heart rate and nasal airflow signals.

Phase	Goodness of Fit (*p*-Value)	
	Heart Rate	Airflow
8 min	0.35	0.74
9 min	0.69	0.91
10 min	0.70	0.55
11 min	0.53	0.53
19 min	0.93	0.58
20 min	0.58	0.99
21 min	0.46	0.64
23 min	0.33	0.65
24 min	0.52	0.12
25 min	0.14	0.69
26 min	0.52	0.84
28 min	0.15	0.30
29 min	0.13	0.67
30 min	0.99	0.82
31 min	0.58	0.31
33 min	0.12	0.67
34 min	0.28	0.85

**Table 6:** Overall assessment of phase-level normality for log-transformed heart rate and nasal airflow signals using Fisher’s method.

Physiological Signal	Phases	Goodness-of-Fit (*p*-Value)	Interpretation
Heart rate	17	0.56	No evidence of departure from normality
Airflow (nasal)	17	0.99	No evidence of departure from normality

**Table 7:** SPC rule violations by signal and sleep stage.

Physiological Signal	Sleep Stage	Phases Evaluated	Phases with ≥1 Rule Violation	Percentage (%)
Heart rate	Wake	8	6	75.0
Heart rate	Non-REM	9	1	11.1
Airflow (nasal)	Wake	8	4	50.0
Airflow (nasal)	Non-REM	9	1	11.1

## Data Availability

The polysomnography (PSG) dataset analyzed in this study is openly available from the Sleep Heart Health Study Polysomnography Database (SHHPSGDB) hosted on PhysioNet. The data can be accessed and downloaded at https://physionet.org/content/shhpsgdb/1.0.0/ (accessed on 7 May 2026) under an open-access license. This resource includes raw PSG signal files and corresponding annotation files such as sleep stages, respiratory events, and heart rate measurements.

## Abbreviations

The following abbreviations are used in this manuscript:

SPC	Statistical Process Control
HDDSA	Health Diagnostic Data Stream Analytics
HIT	Health Information Technology
AHRQ	Agency of Healthcare Research and Quality
IS	Information System
IT	Information Technology
HR	Heart Rate
CL	Centerline
UCL	Upper Control Limit
LCL	Lower Control Limit
CQI	Continuous Quality Improvement
TQM	Total Quality Management
DMAIC	Define, Measure, Analyze, Improve, Control
DMEDI	Define, Measure, Explore, Develop, Implement
CPI	Continuous Product Improvement
NPI	New Product Introduction
KPI	Key Performance Indicator
BOA	Bank of America
ECG	Electrocardiogram
EEG	Electroencephalogram
REM	Rapid Eye Movement
EMG	Electromyogram
EOG	Electrooculogram
PSG	Polysomnography
OSA	Obstructive Sleep Apnea
SHHS	Sleep Heart Health Study
AHI	Apnea–Hypopnea Index
AF	Atrial Fibrillation
CEP	Complex Event Processing
CUSUM	Cumulative Sum
EWMA	Exponentially Weighted Moving Average
AI	Artificial Intelligence
CSA	Central Sleep Apnea
ML	Machine Learning
xAI	Explainable Artificial Intelligence
